# Clinico-Epidemiological Characteristics of Patients With Inflammatory Bowel Disease in Egypt: A Nationwide Multicenter Study

**DOI:** 10.3389/fmed.2022.867293

**Published:** 2022-04-19

**Authors:** Mohamed Elbadry, Mohamed O. Nour, Mohamed Hussien, Elsayed Awad Ghoneem, Mohammed A. Medhat, Hany Shehab, Sherif Galal, Mohamed Eltabbakh, Fathiya El-Raey, Mohamed Negm, Shimaa Afify, Walaa Abdelhamed, Ahmed Sherief, Ahmed Abdelaziz, Mohamed Abo Elkasem, Aya Mahrous, Ghada Kamal, Maha Maher, Omar Abdel-Hameed, Abubakr Elbasuny, Islam El-Zayyadi, Ahmed Bassiony, Abdelmajeed Moussa, Essam Bedewy, Asem Elfert, Mohamed El Kassas

**Affiliations:** ^1^Department of Endemic Medicine, Faculty of Medicine, Helwan University, Cairo, Egypt; ^2^Department of Public Health and Community Medicine, Damietta Faculty of Medicine, Al-Azhar University, Damietta, Egypt; ^3^Faculty of Public Health and Health Informatics, Umm Al-Qura University, Makkah, Saudi Arabia; ^4^Department of Gastroenterology, Hepatology and Infectious Diseases, Kafrelsheikh University, Kafrelsheikh, Egypt; ^5^Department of Hepatology and Gastroenterology, Mansoura University, Mansoura, Egypt; ^6^Department of Tropical Medicine and Gastroenterology, Assiut University, Assiut, Egypt; ^7^Integrated Clinical and Research Center for Intestinal Disorders (ICRID), Department and Endemic Medicine, Cairo University, Cairo, Egypt; ^8^Department of Tropical Medicine, Faculty of Medicine, Zagazig University, Zagazig, Egypt; ^9^Department of Tropical Medicine, Faculty of Medicine, Ain Shams University, Cairo, Egypt; ^10^Department of Hepatogastroenterology and Infectious Diseases, Damietta Faculty of Medicine, Al-Azhar University, Damietta, Egypt; ^11^Department of Gastroenterology, National Hepatology and Tropical Medicine Research Institute, Cairo, Egypt; ^12^Department Tropical Medicine and Gastroenterology, Sohag University, Sohag, Egypt; ^13^Department of Hepatology and Gastroenterology, Talkha Central Hospital, Talkha, Egypt; ^14^Department of Tropical Medicine and Gastroenterology, Faculty of Medicine, Aswan University, Aswan, Egypt; ^15^Department of Tropical Medicine, Faculty of Medicine, Alexandria University, Alexandria, Egypt; ^16^Department of Tropical Medicine and Infectious Diseases, Faculty of Medicine Tanta University, Tanta, Egypt

**Keywords:** ulcerative colitis, Crohn's disease, multicenter study, Egypt, inflammatory bowel disease

## Abstract

**Background and Aims:**

Ulcerative colitis (UC) and Crohn's disease (CD) are the most common types of Inflammatory bowel disease (IBD), with variable responses to traditional therapies and unpredicted prognosis. In Egypt and most developing countries, the lack of recent epidemiological and prognostic data adversely affects management strategies. We collected and analyzed data of patients with IBD from multiple centers across Egypt to evaluate patients' clinical and epidemiological characteristics.

**Methods:**

This retrospective multicenter study included patients diagnosed with IBD between May 2018 and August 2021, at 14 tertiary gastroenterology units across Egypt. Record analysis addressed a combination of clinico-epidemiological characteristics, biochemical tests, stool markers, endoscopic features, histological information, and different lines for IBD treatment.

**Results:**

We identified 1104 patients with an established diagnosis of IBD; 81% of them had UC, and 19% showed CD. The mean age of onset was 35.1 ± 12.5 years ranging from 5 to 88 years, the mean duration of illness at inclusion was 13.6 ± 16.7 years, gender distribution was almost equal with a significant male dominance (60.4%, *p* = 0.003) among patients with CD, 57% were living in rural areas, and 70.5% were from Delta and Coastal areas. Two hundred nineteen patients (19.8%) displayed comorbid conditions, primarily associated with CD. The most frequent complaints were diarrhea (73.2%), rectal bleeding (54.6%) that was significantly higher among patients with UC (64%, *p* < *0.001*), and 46.8% with abdominal pain (more often with CD: 71%, *p* < *0.001*). Conventional therapy was effective in treating 94.7% of patients. The main lesion in patients with CD was ileal (47.8%); patients with UC mainly exhibited proctosigmoiditis (28.4%). Dysplasia was detected in 7.2% of patients, mainly subjects with UC.

**Conclusions:**

To our knowledge, our effort is the first and largest cohort of Egyptian patients with IBD to describe clinical and epidemiological characteristics, and diagnostic and management approaches. More extensive prospective studies are still needed to fully characterize disease distribution, environmental factors, and pathological features of the disease.

## Introduction

In 2017, 6.8 million Inflammatory bowel disease (IBD) cases were reported globally. The prevalence of IBD has increased substantially in many regions of the world, creating a substantial social and economic burden on health systems (1). Ulcerative colitis (UC) and Crohn's disease (CD) are major chronic IBD conditions in addition to indeterminate colitis that cause varying degrees of gastrointestinal (GI) tract inflammation. This classification was addressed by Montreal working party and commonly used in clinical practice and also for future serological and genetic studies in IBD (2). Data from several studies report a relationship between smoking and CD. However, quitting smoking may be accompanied by an increased risk of UC (3). Dietary fiber (particularly vegetables and fruits), saturated fats, sleep disorders, depression, and low vitamin D levels have been associated with increased IBD incidence. Also, stress, microbiota, some medications, such as NSAIDs, and early antibiotic exposure during infancy are factors that might increase the risk for IBD incidence, especially in genetically susceptible individuals (4, 5). Therefore, some studies concluded that, modification of one or more of these environmental factors has a bidirectional effect on the disease activity (6). Disease presentation includes signs and symptoms such as diarrhea, rectal bleeding, and abdominal pain. Fever, weight loss, extraintestinal symptoms, and fatigue may be observed (7). A serious complication of IBD is the malignant transformation of colonic mucosa that increases the incidence of colorectal carcinoma. Further, psychiatric health is negatively affected by IBD, especially in young patients (8). Also, extraintestinal manifestations (EIMs) can occur in some patients and affect joints, the hepatobiliary system, skin, and eyes (9).

Assessment of patients with IBD includes physical examination with a focus on extraintestinal complications, laboratory evaluation [C-reactive protein (CRP), erythrocytes sedimentation rate (ESR), and fecal calprotectin (FC)], endoscopy, and different imaging modalities (10). All these clinical examinations are used to confirm the diagnosis, gauge disease extent, and evaluate severity. Treatment depends on disease extent and severity (7).

The disease is a worldwide concern, but the incidence is highest in the United States, Sweden, and United Kingdom (11–13). IBD has a growing incidence in the Middle East and North Africa; however, a lack of accurate registry and epidemiological cohort studies are still obstacles to evaluating the current situation (14–16). UC is more common than CD in various parts of the world. In Egypt, few data regarding the epidemiology of IBD are available; however, some studies suggest the relative incidence ratio of UC and CD is 6:1 (17).

We conducted this retrospective cross-sectional multicenter study to represent all of Egypt. We collected clinical information to evaluate epidemiological characteristics, laboratory and imaging findings, colonoscopy results, and different treatment strategies. We aimed to assess updated data to draw a real map of IBD in Egypt in order to help us introduce scientific recommendations to optimize IBD diagnosis and management strategies.

## Methods

### Study Design, Settings, and Inclusion Criteria

This retrospective multicenter study was conducted on patients diagnosed with IBD at 14 tertiary GI units. These centers are affiliated with universities, distributed across Egypt, and represent the main geographical areas within the country where most of the Egyptian population is concentrated along the banks of the Nile River and on the river's delta including (1) Greater Cairo (Helwan, Cairo, Ain Shams, Al-Azhar, and the National Hepatology and Tropical Medicine Research Institute), (2) Delta region (Mansoura, Tanta, Zegazeg, and Kafr Elsheikh), (3) Coastal region (Alexandria and Damietta), (4) Upper Egypt (Sohag, Assiut, and Aswan). Patients from remote areas in Egypt, Sinai Peninsula, and Oases are largely referred to these tertiary centers. Data were collected both manually and electronically from available medical records between May 2018 and August 2021. Medical records of patients of all ages and both sexes with a confirmed diagnosis of IBD during the study period were enrolled.

### Study Variables

Demographic features recorded were age of onset, gender, residence, geographic area, duration of illness, history of smoking, associated comorbidities, and other autoimmune diseases. Disease characteristics, including the severity of symptoms, presence of EIMs, type of medical treatment, and history of surgical intervention, were tabulated. Remission was defined as complete resolution of signs and symptoms, and endoscopic and histological healing of colon mucosa.

### Diagnosis of IBD

The diagnosis of CD or UC was based on a combination of clinical manifestations, biochemical tests, stool markers, endoscopic features, and histological evaluation.

The recorded biochemical information included complete blood count (CBC), CRP, ESR, liver, and kidney function tests. Also, FC as a stool marker of intestinal inflammation was noted. Reports of abdominal ultrasonography and upper endoscopy were retrieved for all enrolled patients.

Endoscopic features consistent with UC were continuous and confluent colon inflammation with clear demarcation and rectal involvement. Endoscopic features consistent with CD were discontinuous lesions, mucosal nodularity, ulceration (both aphthous and linear), and strictures. Disease distribution and activity of UC were evaluated using the Montreal classification and Mayo score, respectively. While patients with CD were evaluated according to the severity of the onset of the disease using Harvey-Bradshaw score and Phenotypic distribution according to Montreal classification (2, 18).

### Treatment of IBD

All recorded data about treatment approach either step up or step down, type of treatment either topical or systemic or biological in addition to antibiotic and steroid therapy were extracted from the patients‘ medical records and statistically analyzed.

### Ethical Considerations

All procedures involving human participants were carried out according to the ethical standards of the institutional and/or national research committee and with the 1964 Helsinki declaration and its later amendments, or comparable ethical standards. The study was approved by the Research Ethics Committee for human subject research at the Faculty of Medicine, Helwan University (Serial: 76-2021). The study data set was fully anonymized.

### Statistical Analysis

Analysis used SPSS version 25.0 (IBM SPSS Statistics for Windows, Armonk, NY: IBM Corp., USA). Mean ± SD was used for quantitative variables, and frequency and percentage were used for qualitative variables. Mann-Whitney and Wilcoxon tests were used to assess the differences in means of quantitative non-parametric variables. Chi-square and Fisher's Exact tests were used to assess differences in the frequency of qualitative variables. The statistical methods assumed a significance level of *p* < 0.05 and a highly significant level of *p* < 0.001.

## Results

The study included 1,104 patients with an established diagnosis of IBD. Ulcerative colitis was diagnosed in 81.3% of the study population, while −18.7% exhibited CD. The mean age of onset was 35.1 ± 12.5 years ranging from 5 to 88 years with mean duration of illness at inclusion of 13.6 ± 16.7 years for the cohort. The gender distribution was almost equal; however, a significant male dominance (60.4%, *p* = 0.003) was observed among patients with CD. The residence of the recruited patients was found to be in rural areas in 57% of cases. These patients displayed a higher prevalence of UC (59.2%, *p* = 0.002). Further, 70.5% were from North of Egypt. About of patients suffered from comorbid conditions, mainly hypertension (HTN) (9.1%) and diabetes (DM) (8.4%). Most comorbidities were more frequent among patients with CD, except for DM and other autoimmune diseases (Table 1).

**Table 1:** General characteristics of patients with inflammatory bowel disease in Egypt (May 2018 to August 2021).

**Variables**		**Total *n =* 1,104 (%)**	**UC group *n =* 897 (%)**	**CD group *n =*207 (%)**	**P-value**
Age of onset (years)	Mean ± SD Min–Max	35.1 ± 12.5 5–88	35.3 ± 12.5 5–88	34.1 ± 12.4 9–70	0.250
Gender	Male	561 (50.8)	436 (48.6)	125 (60.4)	0.003^*^
	Female	543 (49.2)	461 (51.4)	82 (39.6)	
Residence	Urban	475 (43.0)	366 (40.8)	109 (52.7)	0.002^*^
	Rural	629 (57.0)	531 (59.2)	98 (47.3)	
Geographic area	Greater Cairo	154 (13.9)	115 (12.8)	39 (18.8)	0.001^*^
	Delta & Coastal region	778 (70.5)	626 (69.8)	152 (73.4)	
	Upper Egypt	172 (15.6)	156 (17.4)	16 (7.7)	
Disease duration at inclusion (years)	Mean ± SD	13.6 ± 16.7	15.1 ± 17.9	12.8 ± 14.7	0.086
Smoking		194 (17.6)	149 (16.6)	45 (21.7)	0.085
Comorbidities*^a^*		219 (19.8)	176 (19.6)	43 (20.8)	0.700
Diabetes mellitus		93 (8.4)	87 (9.7)	6 (2.9)	0.001^*^
Other autoimmune disease*^b^*		36 (3.3)	35 (3.9)	1 (0.5)	0.008^*^
Cardiac diseases		33 (3.0)	15 (1.7)	18 (8.7)	<0.001^*^
Hepatic diseases		35 (3.2)	12 (1.3)	23 (11.1)	<0.001^*^
Renal diseases		24 (2.2)	4 (0.4)	20 (9.7)	<0.001^*^
Hypertension		101 (9.1)	73 (8.1)	28 (13.5)	0.022^*^
Others*^c^*		23 (2.1)	20 (2.2)	3 (1.4)	0.598

*UC, Ulcerative colitis; CD, Crohn's disease*.

a *Some patients have more than one comorbid condition*.

b *For Ulcerative colitis; 6 hypothyroidism, 5 SLE, 5 thyroiditis, 5 rheumatoid arthritis, 3 ankylosing spondylitis, 2 vitiligo, 2 autoimmune hepatitis, panhypopituitarism, axial arthralgia, scleroderma, myositis, polyarthritis, Behcet disease, and autoimmune hemolytic anemia*.

*For Crohn's disease; Eosinophilic gastritis*.

c *For Ulcerative colitis; 3 TB, 2 hemorrhoids, 2 ulcers, perforation/sigmoid colon mass, steroid-dependent, fistula (colonic), depression, FMF, 1ry infertility, hyperthyroidism, epilepsy, chest disease, pyoderma gangrenosum, G6PD deficiency, pityriasis versicolor, and conjunctivitis*.

*For Crohn's disease; osteoporosis, TB, DVT, and Marjolin ulcer*.

* *Significant*.

### Clinical Presentations at Diagnosis

The most frequent manifestations, at the time of diagnosis, among all patients was diarrhea (73.2%) which was similar for both UC and CD. Bowel movements/day was 4.7 ± 2.2 ranging from 0 to 20. -Rectal bleeding was reported by 54.6% of patients, and it was significantly higher among UC subjects, while 48.6% -of patients complained from abdominal pain. Abdominal pain was higher among patients with CD, but the difference was insignificant. Weight loss was seen in 15.3% of patients, and 11.7% displayed EIM, mainly arthropathy. Fever was the least frequent symptom (8.5%) and was significantly higher among subjects with CD (Table 2).

**Table 2:** Clinical characteristics of patients with inflammatory bowel disease in Egypt (May 2018 to August 2021).

**Variables**		**Total *n =* 1104 (%)**	**UC group *n =* 897 (%)**	**CD group *n =*207 (%)**	***P*-value**
Bowel motions /day	Mean ± SD Min–Max	4.7 ± 2.2 0–20	4.6 ± 2.0 0–12	5.0 ± 2.8 1–20	0.162
Pulse	Mean ± SD Min–Max	83.7 ± 12.5 60–133	84.0 ± 12.8 60–133	83.0 ± 11.6 66–120	0.234
Diarrhea		808 (73.2)	655 (73.0)	153 (73.9)	0.862
Rectal bleeding		603 (54.6)	574 (64.0)	29 (14.0)	<0.001^*^
Abdominal pain		517 (46.8)	370 (41.2)	147 (71.0)	<0.001^*^
Fever		94 (8.5)	66 (7.4)	28 (13.5)	0.008^*^
Extraintestinal manifestations*^a^*		129 (11.7)	107 (11.9)	22 (10.6)	0.719
Significant weight loss		169 (15.3)	130 (14.5)	39 (18.8)	0.133

*UC, Ulcerative colitis; CD, Crohn's disease*.

a *For Ulcerative colitis (some patients have more than one condition); 43 arthropathy, 10 pyoderma gangrenosum, 9 oral aphthous ulcers, 7 sacroiliitis, 7 primary sclerosing cholangitis, 6 episcleritis, 4 gall bladder stones, 4 uveitis, 3 erythema nodosum, 3 skin lesions, 3 cholangitis, 3 ankylosing spondylitis, Celiac disease, sickle cell anemia, Budd-Chiari syndrome, DVT and thrombosis, iridocyclitis, renal stones, and fistula*.

*For Crohn's disease (some patients have more than one condition); 14 arthropathy, 3 skin lesions, 2 erythema nodosum, 2 sacroiliitis, 2 oral aphthous ulcers, gall bladder stones, ankylosing spondylitis, Celiac disease, retroperitoneal fibrosis, and retinitis*.

* *Significant*.

### Laboratory Investigations at Time of Diagnosis

Laboratory characteristics at the time of diagnosis showed significantly improved HB, CRP, ESR, WBC, and FC levels after treatment in both UC and CD patients (Table 3). PLT and AST were decreased in CD and UC patients, respectively. No notable change was observed in serum levels of total protein, albumin, and ALT.

**Table 3:** Laboratory characteristics of patients with inflammatory bowel disease in Egypt (May 2018 to August 2021) before and after treatment.

**Variables**	**UC group**		**P-value**	**CD group**		**P-value**
	**Before**	**after**		**Before**	**After**	
WBC	7.9 ± 3.2	6.5 ± 2.2	<0.001^*^	7.7 ± 3.3	6.2 ± 2.0	<0.001^*^
HB	11.2 ± 1.9	11.9 ± 1.6	<0.001^*^	11.8 ± 1.8	12.3 ± 1.4	0.003^*^
PLT	314.4 ± 118.7	308.3 ± 119.0	0.199	284.5 ± 125.9	231.6 ± 125.2	0.001^*^
Total Protein	6.97 ± 0.79	7.09 ± 0.70	0.514	7.08 ± 1.13	7.24 ± 1.24	0.361
Albumin	3.76 ± 0.59	3.65 ± 0.55	0.404	3.73 ± 0.56	3.72 ± 0.45	0.449
ALT	28.2 ± 19.7	29.8 ± 26.1	0.433	29.0 ± 17.8	24.8 ± 8.8	0.084
AST	30.2 ± 18.6	27.3 ± 15.0	<0.001^*^	25.9 ± 12.1	26.5 ± 10.4	0.465
CRP	21.2 ± 29.9	8.2 ± 20.4	<0.001^*^	34.1 ± 35.7	4.6 ± 6.9	<0.001^*^
ESR	46.2 ± 28.8	25.7 ± 21.7	<0.001^*^	52.9 ± 28.5	21.8 ± 18.5	<0.001^*^
Fecal calprotectin	584.5 ± 652.1	220.1 ± 311.0	<0.001^*^	505.1 ± 712.2	176.5 ± 240.3	<0.001^*^

*WBC, White blood cells; HB, Hemoglobin; PLT, Platelets; ALT, Alanine aminotransferase; AST, Aspartate aminotransferase; CRP, C reactive protein; ESR, erythrocyte sedimentation rate*.

* *Significant*.

### Imaging, Endoscopic, and Histopathological Features of the Rectruited Patients

**Figure 1:**
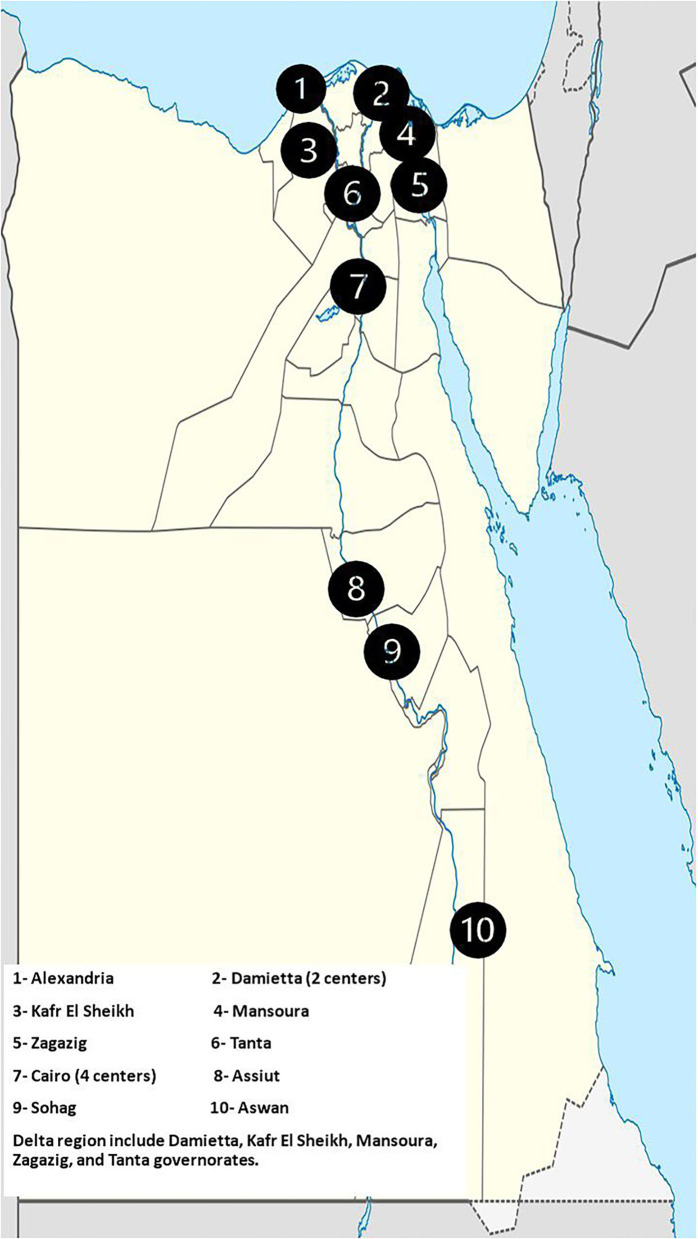
Distribution of the participating centers in the study across Egypt.

patients with patients with The lesions in CD subjects at diagnosis were mainly ileal (47.8%) and ileocolonic (38.6%). Lesions were mainly non-stricturing and non-penetrating (61.4%) and less commonly stricturing (20.8%) (Figure 1).

**Figure 2:**
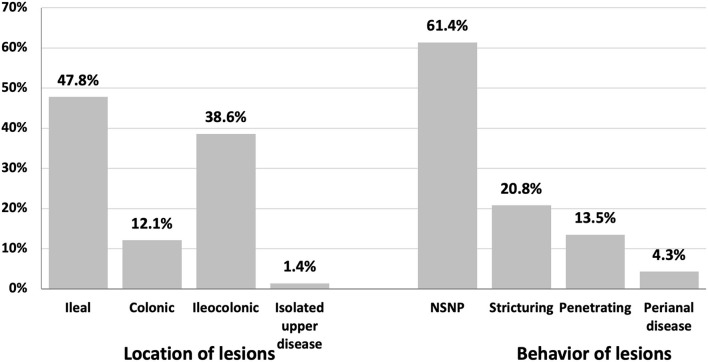
Location and behavior of lesions by colonoscopy in patients with Crohn's disease in Egypt (May 2018 to August 2021) at the time of diagnosis.

At diagnosis, CT/MRI revealed fistulization in about 11.1% of patients, and no abnormality was detected in about 18.4%. Colonoscopy showed proctosigmoiditis in 28.4% of patients with UC, proctitis in 25.1%, and pancolitis in 22.9%. Mild, moderate, and severe lesions were detected in 44.7, 35.8, and 19.5%, respectively (Figure 2).

Dysplasia was detected histologically in 80 patients (7.2%), mainly associated with UC (75 patients). Low-grade dysplasia was predominant, seen in 68 patients (6.2%). Sixty-four patients were diagnosed with UC (*p* = 0.012).

### Lines of Management for the Recruited Patients

Most patients (94.7%) were managed using a step-up approach. The main antibiotics used were quinolone and metronidazole (40–50%). Topical therapies available in Egypt are (5-ASA suppositories and budesonide enema, and they) were used in 21–31% of cases. Unfortunately, other forms of topical therapies (5-ASA enemas, 5-ASA foams, 5-ASA gels, Corticosteroids foams, tacrolimus suppositories, and cyclosporin enemas) are not present in Egypt. Systemic treatment was prescribed for 50–86%, mainly with 5-ASA. About one-fourth received biological treatment, and only 2.6% were referred for coloniccolectomy or intestinal resection and anastomosis (Table 4).

**Table 4:** Treatment characteristics of patients with inflammatory bowel disease in Egypt (May 2018 to August 2021).

**Treatment*^a^***		**Total *n =* 1104 (%)**	**UC group *n =* 897 (%)**	**CD group *n =*207 (%)**
**Approach** **for treatment**	Step-up	1045 (94.7)	864 (96.3)	181 (87.4)
	Step-down	59 (5.3)	33 (3.7)	26 (12.6)
**Antibiotics**			
Metronidazole		442 (40.0)	366 (40.8)	76 (36.7)
Quinolone		556 (50.4)	478 (53.3)	78 (37.7)
Others		60 (5.4)	40 (4.5)	20 (9.7)
**Local**			
5-ASA		344 (31.2)	335 (37.3)	9 (4.3)
Steroids		239 (21.6)	230 (25.6)	9 (4.3)
**Systemic**			
5-ASA		955 (86.5)	797 (88.9)	158 (76.3)
Steroids		691 (62.6)	582 (64.9)	109 (52.7)
Azathioprine		553 (50.1)	438 (48.8)	115 (55.6)
**Biological*^b^***		281 (25.5)	221 (24.6)	60 (29.0)
**Surgical** (resection)		29 (2.6)	14 (1.6)	15 (7.2)

*UC, Ulcerative colitis; CD, Crohn's disease; 5-ASA, 5-aminosalicylic acid*.

a *Some patients received more than one treatment regimen and this treatment represented the cumulative frequency of drug usage*.

b *Some patients received more than one type of biological treatment*.

## Discussion

This study is the largest cohort evaluated for IBD in Egypt to the best of our knowledge. We found that the prevalent cases of UC are about four-fold greater than CD. This ratio is higher than reported elsewhere. In 2009, census data estimated that 1,171,000 Americans exhibited IBD (565,000 CD and 593,000 UC) (19). In 2018, the overall prevalence of IBD, CD, and UC in the UK were 725, 276, and 397 per 100 000 people, respectively (20). The occurrence of bleeding, which is more common in UC, is a potent stimulant for seeking medical advice, and so UC cases can be identified more frequently than CD cases. Furthermore, the diagnosis of CD may require exhaustive workup including detailed history taking, laboratory investigations, imaging modalities, and colonoscopy with ileal intubation or even enterostomy. These diagnostic facilities are expensive (not fully covered by insurance) and not available in all centers, particularly that more than half of our study population are from rural areas. However, such differences may be due to many factors that should be studied in community-based research. Key issues may be socioeconomic status, environmental conditions, and access to diagnostic and treatment facilities.

Our study's mean age of onset was 35 and 34 years for UC and CD subjects, respectively. The onset of IBD in adults was reported as 31–34 years in North America, Western Europe, and Oceania (21–25). In Asia, the median age at diagnosis of CD was 34 years, and for UC was 42 years. UC was also found to start at more advanced ages, up to 79 years (26, 27).

The development of UC in our cohort was independent of gender, and however, CD was more common in males. These findings are consistent with epidemiological data from Europe, North America, and Oceania (21–25). However, UC was more common in males in data from Asia (28).

More than half of the study population was from rural areas, inconsistent with the available literature on IBD demographics. The urban population in Egypt has been almost stable since 2010 and represents 42.8% of the Egyptian population (29). However, the lifestyle in rural areas has been urbanized, and this change should be studied selectively. Zuo et al. (30) indicated that rapid urbanization in the developing world is associated with an increasing incidence of several autoimmune diseases, including IBD. Urbanization impacts gut microbiota through westernization of diet, raised pollution levels, increased usage of antibiotics, and better hygiene status. A westernized diet is low in carbohydrates and high in animal proteins and fats. This diet will alter gut microbiota.

The clinical presentation of IBD in our cohort is consistent with globally published data. The predominant manifestations of UC were diarrhea, rectal bleeding, and mucous discharge from the rectum (31). Conversely, CD was characterized by prolonged intermittent diarrhea with abdominal pain (32–35).

Ileal (47.8%) and ileocolonic (38.6%) regions were predominant sites of CD lesions, Colonic lesions were seen in (12.1%), while isolated upper GI CD was detected in only 1.4% of our study population. Slightly less than two-thirds of our patients (61.4%) showed non-complicated disease. Twenty-point eight percent exhibit stricturing CD, and 13.5% show penetrating CD. These data are consistent with data from Europe and North America, where 27–42% of patients with CD have ileal lesions at the time of diagnosis, 23–33% exhibit ileocolonic disease, 28–35% show colonic lesions, and only 1–6% of patients present with upper GI CD (24, 36–39). Data from stricturing CD is estimated to occur in 29% and 19% of patients with CD in Europe (38) and North America (36), respectively. Conversely, Asian cohorts reported that more than half of patients with CD have an ileocolonic disease (40–42).

Multiple serum biomarkers were evaluated for their ability to confirm the IBD disease type and to predict the disease course. The atypical antineutrophil cytoplasmic antibody (atypical pANCA) and anti-Saccharomyces cerevisiae antibody (ASCA) are the most studied antibodies in IBD patients. is most often expressed by patients with ulcerative colitis. Atypical pANCA is more common patients in patients with UC while ASCA in more frequent in CD patients (43). However, these tests are expensive and covered by health insurance, so they are commonly tested in Egypt.

**Figure 3:**
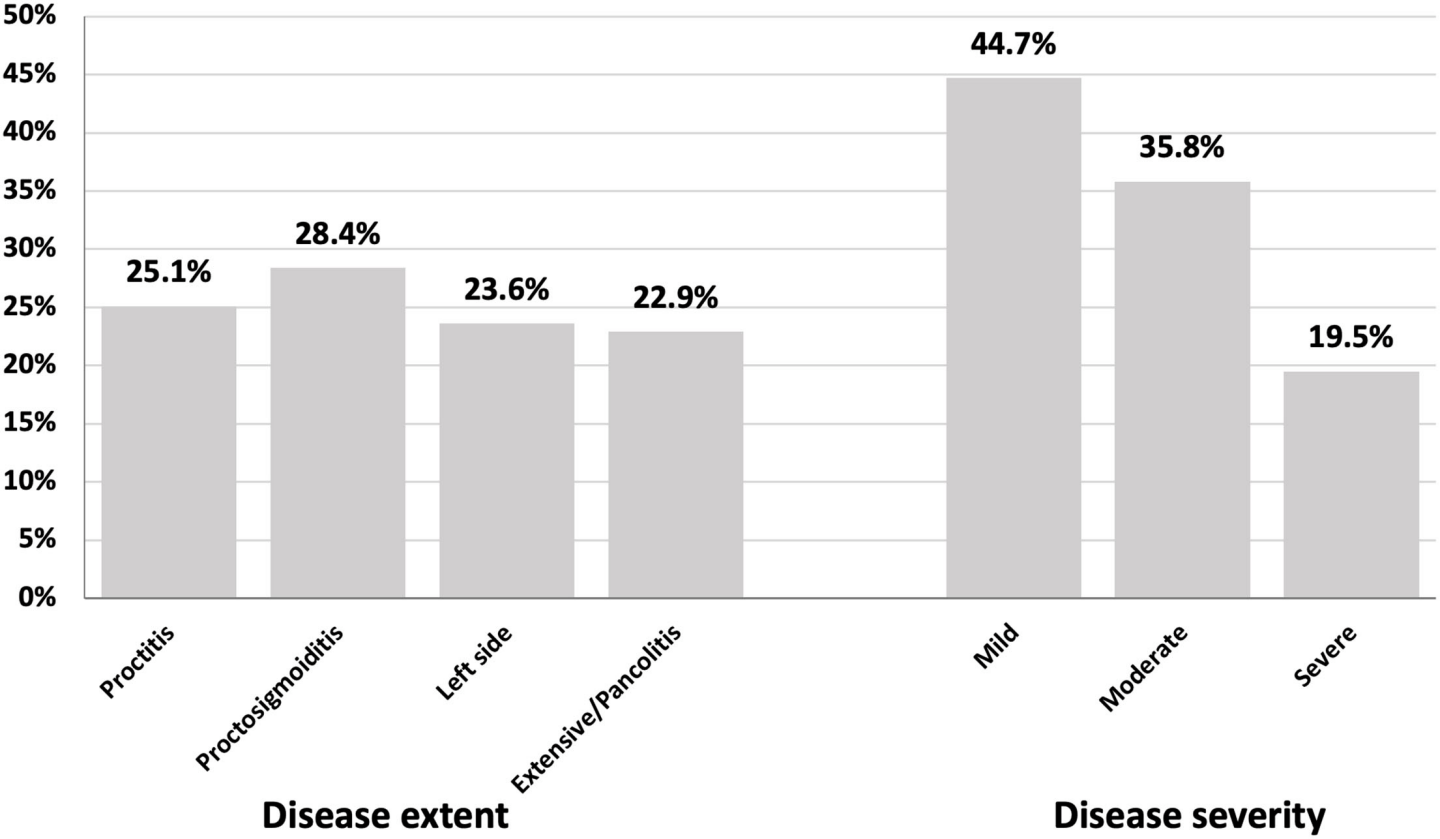
Disease extent and severity by colonoscopy in patients with ulcerative colitis in Egypt (May 2018 to August 2021).

Proctosigmoiditis was the most common disease site in UC cases in our cohort. Proctitis, left-sided colitis, and pancolitis were also seen in order of descending incidence. Most cases were mild to moderate at diagnosis. Disease extent and severity by colonoscopy in patients with ulcerative colitis are shown in Figure 3. Other cohorts showed that left-sided colitis was the most common disease site in Europe (39, 44).

Most of our patients (96.3% of patients with UC and 87.4% of patients with CD) received conventional medical therapy as a first-line treatment even if they were indicated for biological therapy. The high costs of biological therapy likely limit its use, which may not be sustainable. However, health insurance and government-sponsored treatment have started to cover the costs of biological therapy at many centers in Egypt. Infliximab and adalimumab began to be used in 2013, and new biological therapies, such as ustekinumab have been used more recently (45).

Fortunately, conventional step-up therapy is a practical approach for treating Egyptian patients with IBD. Significant improvement in inflammatory markers, including CRP, ESR, and FC, were observed after treatment of both UC and CD.

Colorectal cancer is the most serious complication for patients with IBD, and dysplasia usually precedes the development of colorectal cancer (46). In our study, cases with confirmed dysplastic lesions constituted 7.2% of the total population. Most were low-grade and discovered on the first diagnosis.

Available evidence suggests that IBD incidence in Africa is increasing. Data are still lacking to understand the disease pattern across this continent. A few reports from North and South Africa are available (47).

The major strengths of our study are its large sample size and geographic diversity of patients, and collected data provide a comprehensive, updated picture of IBD in Egypt. Data describe differences in some clinical, epidemiological aspects of the disease. However, the study has some limitations. First, the distinction between UC and CD was not always clear. We depended on the most likely diagnosis from the treating physician. Lacking the distinction between IBD subtypes is a global problem. In a recently published population-based cohort study in European countries including 1,289 patients with IBD, the confirmation of IBD subtype was impossible in 7% of the study population, even after 5 years of follow-up (48). Second, the incomplete data for the follow-up of some patients did not allow us to track the changes in the disease patterns or to do a statistical analysis to see the histological features at time diagnosis will affect the outcome of our patients. Third, we may have missed early mild cases of IBD, especially in the private sector. Fourth, risk factors and environmental determinants of IBD were not discussed. Finally, certain socioeconomic factors, such as income and education, in addition to the yearly incidence of new cases could not be assessed.

## Conclusion

To our knowledge, our study is the first Egyptian cohort study from multiple highly specialized GI centers from all over the country. It examines the largest cohort of Egyptian patients with IBD and describes the clinical, epidemiological presentation, diagnostic procedures, disease behavior, and prognostic implications along with available therapeutic options. The number of IBD cases was higher in rural than urban areas despite limited resources and relatively poor facilities in rural areas. More effort should be directed toward screening patients with IBD in rural areas for early detection and proper management of the disease. Such effort may relieve the burden of unexpected serious maladies. Step-up conventional therapy for patients with IBD is still recommended and effective, especially in countries with limited resources. More extensive prospective epidemiological studies in Egypt, other countries of the Middle East, and Africa are still needed to fully characterize disease distribution, environmental factors, and pathological features of IBD. Such data can be compared with other parts of the world to complete the global map of IBD and produce worldwide guidelines for managing this severe expanding disease.

## Data Availability Statement

The raw data supporting the conclusions of this article will be made available by the authors, without undue reservation.

## Ethics Statement

The studies involving human participants were reviewed and approved by Research Ethics Committee for human subject research at the Faculty of Medicine, Helwan University. Written informed consent for participation was not required for this study in accordance with the national legislation and the institutional requirements.

## Author Contributions

MElb and MEK: study design. MON: data analysis. MElb, MON, MH, EG, MAM, HS, SG, MElt, FE-R,MN, SA,WA, AS, AA, MA, AMa, GK, MM, OA-H, AE, IE-Z, AB, AMo, EB, and AE: patient recruitment, data collection, and writing up of the first draft of the paper. All authors revised and approved the final version of the manuscript.

## Conflict of Interest

The authors declare that the research was conducted in the absence of any commercial or financial relationships that could be construed as a potential conflict of interest.

## Publisher's Note

All claims expressed in this article are solely those of the authors and do not necessarily represent those of their affiliated organizations, or those of the publisher, the editors and the reviewers. Any product that may be evaluated in this article, or claim that may be made by its manufacturer, is not guaranteed or endorsed by the publisher.
